# Isolated Leptomeningeal Carcinomatosis in Urothelial Carcinoma: A Case Report of Positive CSF Cytology With Negative Imaging

**DOI:** 10.1155/crom/8889550

**Published:** 2025-09-25

**Authors:** Christina Lim, Eric Winquist, Mariamma Joseph, Prathana Nathan

**Affiliations:** ^1^Schulich School of Medicine & Dentistry, Western University, London, Ontario, Canada; ^2^Department of Oncology, Western University and London Health Sciences Centre, London, Ontario, Canada; ^3^Department of Pathology and Laboratory Medicine, Western University and London Health Sciences Centre, London, Ontario, Canada; ^4^Department of Medicine, Western University and London Health Sciences Centre, London, Ontario, Canada

**Keywords:** bladder cancer, leptomeningeal carcinomatosis, urological oncology

## Abstract

Bladder cancer is one of the most common cancers worldwide; however, leptomeningeal carcinomatosis (LMC) is an uncommon and understudied complication. We report a male patient diagnosed with LMC while in apparent clinical remission of metastatic urothelial carcinoma of the bladder. He presented with headaches, vision changes, hearing impairment, and leg weakness. Imaging of the brain and spinal cord was negative; however, the cerebrospinal fluid was positive for malignant cells consistent with high-grade urothelial carcinoma, confirming the diagnosis of LMC. Conservative measures and whole-brain radiation were ineffective, and he died 2 months after his LMC diagnosis. Literature review identified 45 cases of LMC due to bladder cancer, and we summarize these data. It is unclear whether new and more effective systemic treatment approaches for urothelial carcinoma will also reduce the risk of LMC or provide better treatment for LMC, supporting a need for more study of this devastating complication.

## 1. Introduction

Leptomeningeal carcinomatosis (LMC) describes the spread of cancer cells to the leptomeninges, including the pia, arachnoid mater, and the subarachnoid space [1, 2]. LMC is being observed more frequently as the survival of cancer patients increases [3]. It is most commonly seen in primary cancers of the lung (9%–25%), melanoma (6%–18%), non-Hodgkin lymphoma (5%–10%), and breast (5%–8%) but is uncommon with genitourinary (GU) cancers (0.03%) [4, 5]. Bladder cancer is the ninth most common cancer worldwide, with over 330,000 cases diagnosed and causing over 30,000 deaths each year [6]. Urothelial carcinoma constitutes the majority of bladder cancers. We report the case of a patient with metastatic urothelial cancer of the bladder in apparent complete clinical remission presenting with LMC.

## 2. Case

**Figure 1:**
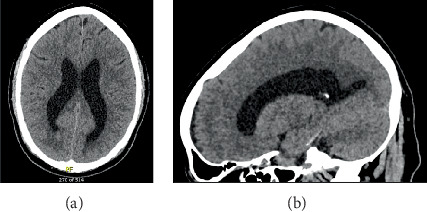
CT brain imaging demonstrating prominent ventricles. (a) Axial and (b) sagittal noncontrast CT images of the brain show prominent lateral and third ventricles without evidence of hydrocephalus, mass effect, or acute intracranial pathology. No signs of leptomeningeal disease were detected radiographically, despite the patient's neurologic symptoms (original image, Department of Medical Imaging, Western University).

A 62-year-old male with a history of metastatic urothelial cancer presented to the emergency department with a 3-day history of occipital headache worse when sitting. He subsequently developed hearing impairment, photophobia, nausea, vomiting, and intermittent left leg weakness resulting in a fall. Computed tomography (CT) of the brain showed prominent ventricles but no other abnormal findings, and he was admitted to the hospital for investigation (Figure 1).

**Figure 2:**
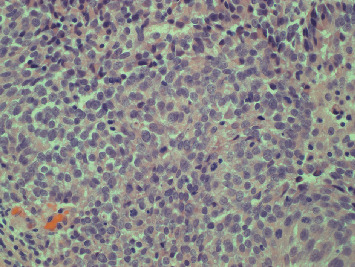
Histopathology of bladder tumor demonstrating invasive high-grade urothelial carcinoma. Hematoxylin and eosin (H&E) stained section of bladder curettings at 40× magnification shows invasive high-grade urothelial carcinoma with marked nuclear atypia and disorganized architecture. There was evidence of muscle invasion (original image, Department of Pathology, Western University).

Seven months earlier, after presenting with hematuria, he underwent cystoscopy, which revealed a significant tumor in the right bladder wall. Transurethral resection of bladder tumor (TURBT) was performed, and the pathology confirmed muscle-invasive (T2), Grade 3 urothelial carcinoma (Figure 2). CT of the thorax, abdomen, and pelvis showed no pelvic lymphadenopathy or visceral metastases but identified a 2.7 cm right bladder wall thickening and several sclerotic densities in the bone. Bone scintigraphy demonstrated sclerotic foci in the L4 vertebral body, left acetabulum, and left inferior pubic ramus, consistent with metastases. The serum alkaline phosphatase level was 72 U/L (upper limit of normal 129 U/L), and prostatic-specific antigen level was within normal limits at 1.94 *μ*g/L.

The patient had received four cycles of gemcitabine (1000 mg/m^2^ Days 1 and 8) and cisplatin (70 mg/m^2^ Day 1) given intravenously every 21 days. This was well tolerated with mild fatigue, dizziness, and tinnitus and complicated by deep venous thrombosis requiring anticoagulation. Following chemotherapy, an FDG PET scan showed no evidence of FDG-avid disease. There was no abnormal uptake in the sclerotic bone lesions, which had regressed, suggesting a response to chemotherapy. The patient was subsequently treated with radiation therapy to the prostate and bladder (55 Gy in 20 fractions) and concurrent chemotherapy with intravenous 5-FU and mitomycin C. He developed a mild COVID-19 infection at the end of his treatment, but otherwise, treatment was well tolerated. Planned avelumab maintenance immunotherapy was preempted by the development of a headache.

Past medical history included a diagnosis of high-grade, noninvasive, urothelial carcinoma of the bladder 7 years earlier treated with TURBT and 10 cycles of BCG, with surveillance cystoscopies for three subsequent years negative for recurrence. He was a lifelong nonsmoker and consumed two drinks of alcohol per day. There was no family history of malignancy.

**Figure 3:**
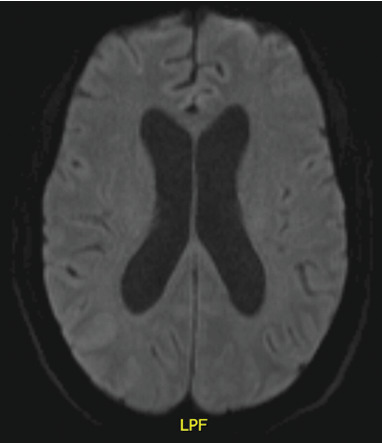
MRI of the brain (DWI-DTI) showing no evidence of neoplastic involvement. Axial diffusion–weighted and diffusion tensor imaging (DWI-DTI) sequences demonstrate no definite hydrocephalus or ventricular obstruction. There is no radiologic evidence of cranial neoplastic disease or leptomeningeal enhancement, despite positive cerebrospinal fluid cytology for malignant cells (original image, Department of Medical Imaging, Western University).

**Figure 4:**
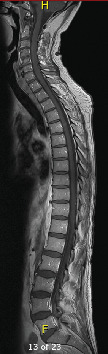
MRI of the entire spine showing no evidence of leptomeningeal carcinomatosis. Sagittal T1-weighted postcontrast MRI of the cervical, thoracic, and lumbar spine shows no abnormal leptomeningeal enhancement or evidence of spinal metastases. Findings were radiographically unremarkable despite positive cerebrospinal fluid cytology, underscoring the diagnostic limitations of imaging in leptomeningeal carcinomatosis (original image, Department of Medical Imaging, Western University).

Following the development of his occipital headaches, lumbar puncture (LP) yielded an opening pressure of 43 cm of water, and cerebrospinal fluid (CSF) analysis showed glucose 0.6 mmol/L, protein 457 mg/L, 47 × 10^6^/L nucleated cells (50% monocyte and 50% lymphocytes), and 19 × 10^6^/L erythrocytes. He received empirical treatment for bacterial and viral meningitis. There was temporary resolution of headache following LP. A repeat LP yielded similar results to the first, and CSF cultures on both were negative. MRI of the brain and spinal cord identified no evidence of hydrocephalus, ventricular obstruction, or neoplastic disease (Figures 3 and 4). Two tiny foci of encephalomalacia were seen in the periphery of the right cerebellar hemisphere indicative of remote infarction. A few days after admission, the patient had an unwitnessed fall followed by a period of reduced level of consciousness raising suspicion for a seizure. CT head showed no acute changes. He was treated with levetiracetam prophylactically. Electroencephalogram did not show evidence of seizure disorder.

**Figure 5:**
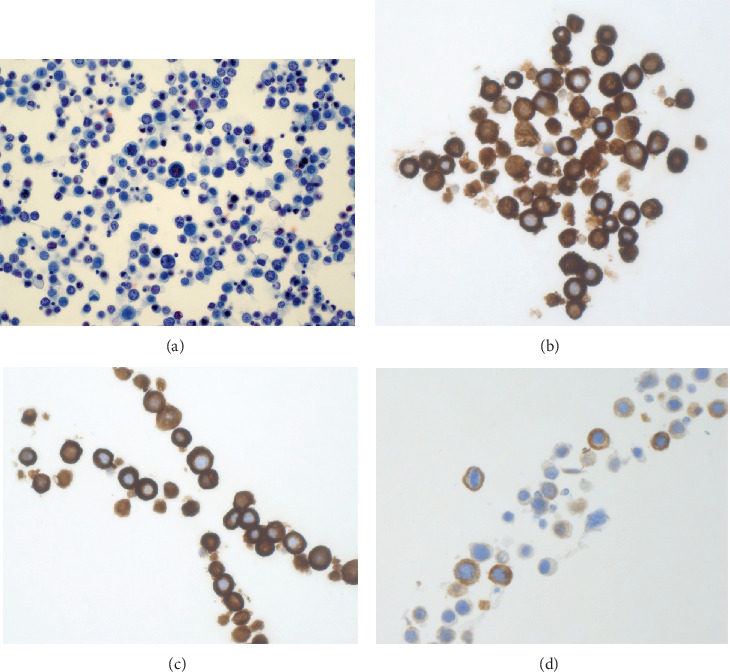
Cerebrospinal fluid cytology and immunocytochemistry confirming metastatic urothelial carcinoma. (a) Papanicolaou-stained cytology smear (40×) of cerebrospinal fluid demonstrates dissociated and loosely cohesive of malignant epithelial cells consistent with high-grade urothelial carcinoma. Immunocytochemistry (40×) shows carcinoma cells positive for (b) cytokeratin AE1/AE3, (c) cytokeratin 7 (CK7), and (d) cytokeratin 20 (CK20), supporting urothelial origin (original image, Department of Pathology, Western University).

CSF cytology was positive for epithelial malignancy consistent with high-grade urothelial carcinoma (Figure 5). The morphologic similarity of the carcinoma cells to those observed in the bladder tissue biopsy, along with dual expression of CK7 and CK20 by immunohistochemistry, confirmed the diagnosis of urothelial carcinoma. The patient was diagnosed with LMC, and an Ommaya reservoir was placed to allow regular CSF drainage to manage persistently increased intracranial pressure. Treatment with dexamethasone and acetazolamide was initiated. Whole-brain radiation was given (2000 cGy in five fractions). Following the suspected seizure, the patient remained bedbound, unable to ambulate, and incontinent. He remained difficult to arouse for the majority of the day, waking only 4–5 h/day. As there was not bulky LMC, hydrocephalus, or parenchymal brain metastases, intrathecal chemotherapy with methotrexate was considered but was deemed futile due to the patient's poor functional status and intractably high CSF pressures. Following two more weeks in the hospital and goals of care discussion with family, the patient was transferred to hospice for end-of-life care and died 1 month later.

## 3. Discussion

To date, 45 cases of LMC from bladder cancer have been reported [7–9]. These cases, including the current case, have been summarized in Table 1. Across the 45 published reports, headache, cranial nerve deficits (e.g., diplopia and hearing loss), and gait disturbances were the most common initial presentations. Over 90% of cases occurred in male patients, and approximately 76% demonstrated positive central nervous system (CNS) imaging, while about 24% (11 of 45 cases) had negative imaging but positive CSF cytology, as in our patient. The median survival across these cases was ~35 days, underscoring the aggressive course of LMC despite treatment efforts.

LMC is thought to be caused by the infiltration of cancer cells into the CSF, then the subarachnoid space, leading to the dissemination and seeding of the leptomeninges [37]. A longer duration of primary cancer has been associated with a higher prevalence of LMC due to an increased likelihood of CNS metastases development and the potential for the CNS to act as a repository for cancer subtypes. As many systemic therapies for cancer do not penetrate the blood–brain barrier (BBB) as effectively, the risk of CNS metastases may be increased, often referred to as a “sanctuary site” effect [1, 2]. This risk may also be increased in patients with extensive prior treatment due to increased permeability of the BBB caused by chemotherapy [35]. LMC is usually accompanied by synchronously progressive primary or metastatic sites (66.6%) rather than being the sole site of metastatic progression as in our patient (16.7%) [7]. Our patient had bone metastases, which are a poor prognostic factor, less responsive to systemic therapy, and possibly a factor in our patient's pattern of recurrence.

LMC can have a variety of initial clinical presentations including, but not limited to, cranial nerve deficits, radicular pain, headache, back pain, visual disturbances, gait disturbances, diplopia, hearing loss, onset of psychiatric disorders, seizures, or cauda equina syndrome [1, 2]. Thus, a high index of suspicion with any neurological signs and symptoms is important in the early recognition of LMC. The most frequently reported symptoms of LMC from bladder cancer include headache, cranial nerve deficits (such as diplopia, hearing loss, or facial droop), and gait disturbances, consistent with our patient's presentation of headache, hearing loss, and limb weakness.

As seen in Table 1, most cases of LMC due to bladder cancer are caused by urothelial carcinoma. However, LMC with other less common histologic types of bladder cancer, including adenocarcinoma and neuroendocrine cancers, has been reported. LMC appears more prevalent in males, with only three patients being female out of the 37 cases that reported the sex of the patient.

The gold standard for diagnosing LMC is the presence of cancer cells on CSF cytology, supplemented by T1-weighted cranial and spine MRI with gadolinium contrast [1, 37–39]. It is not uncommon for the diagnosis to be made by CSF cytology only, MRI only, or both [7–9]. Interestingly, it is more common to have imaging findings of LMC rather than solely CSF findings.

Of the 45 cases summarized in the literature, approximately 24% (11 of 45) demonstrated negative CNS imaging with positive CSF cytology. This emphasizes the importance of considering LMC when brain and spine imaging are unremarkable and supports the use of CSF cytology for definitive diagnosis when clinical suspicion is high. MRI has shown findings of LMC more commonly than CT, likely indicating that MRI is more specific in the diagnosis of LMC. We did not identify other reports of LMC in urothelial cancer patients without evidence of other metastases and negative CNS imaging.

The prognosis of LMC from urothelial carcinoma is poor, with a median survival time of 35 days following diagnosis even with the use of aggressive intervention [1, 7, 40]. Factors such as younger age, better systemic control, higher performance scores (Karnofsky performance score above 70), and a lack of neurological deficits are associated with a better prognosis [1].

There is no standardized treatment approach for LMC [2, 7]. Treatment is complicated by the impermeability of the BBB for many conventional systemic chemotherapy drugs. In addition to measures to treat symptoms and reduce intracranial pressure, the current recommended approach involves systemic management and whole-brain radiation therapy to achieve palliation and prolonged survival [3, 4, 32]. As in this case, ventricular reservoir placement can help relieve neurological symptoms [3]. The data supporting the effectiveness of intrathecal chemotherapy is limited in adult solid tumor patients, but the agent methotrexate may provide temporary benefit in selected patients.

HER2-targeted agents in breast cancer and immunotherapy in melanoma patients with CNS disease have shown greater benefits. Systemic therapy for metastatic urothelial cancer now incorporates immunotherapy and the nectin-targeted antibody-drug conjugate, enfortumab vedotin [41]. Antibody-drug conjugates targeting HER2 and tyrosine kinase inhibitors targeting altered fibroblast growth factor receptors are also active in urothelial cancer. However, information is sparse about whether these new treatments can improve outcomes in patients with LMC or reduce its occurrence.

## 4. Conclusion

Our case report is unique because it involved negative neuroimaging and positive CSF cytology in a patient in apparent clinical remission without synchronous metastatic progression. This demonstrates the potential virulence of metastatic urothelial cancer with an abrupt presentation of LMC. Hopefully, improvements in systemic therapy for urothelial cancer will reduce the incidence of this devastating condition and provide better treatment options. A high index of suspicion for LMC in oncology patients with nonspecific CNS symptoms, along with prompt initiation of CSF cytologic studies and standardization of LMC management, remains important.

## Figures and Tables

**Table 1:** Summary of reported cases of leptomeningeal carcinomatosis in patients with bladder cancer. Overview of 45 published case reports describing patient characteristics, clinical presentation, imaging findings, cerebrospinal fluid (CSF) results, treatment approaches, and outcomes in individuals diagnosed with leptomeningeal carcinomatosis secondary to urothelial carcinoma. Data were extracted from peer-reviewed case reports identified through a structured literature review.

**Author**	**Year of LMC diagnosis**	**1° Ca**	**Age**	**Sex**	**Presenting symptoms of LMC**	**CT**	**MRI**	**CSF**	**Treatment**	**Survival**
Bloch et al. [10]	1986	TCC	67	M	A, HA, and C	+	X	+	WBRT for initial brain mass	4 months
Bishop et al. [11]	1990	TCC	60	M	Sz and Sc	−	X	+	ITM and WBRT	2 months
Bishop et al. [11]	1990	TCC	55	M	NR, C, Da, and Sz	−	X	+	None	< 1 month
Bowen [12]	2010	TCC	50	M	Da, CN, FD, HL, and A	−	+	+	WBRT	2 months
Bruna et al. [13]	2003	TCC	66	M	Sz	−	−	+	ITM	2 months
Butchart et al. [14]	2010	TCC	58	M	PW and Di	−	−	+	None	< 1 month
Cozzarini et al. [15]	1999	TCC	46	M	N, V, HA, and C	−	+	X	ITC	5 months
Cozzarini et al. [15]	1999	TCC	40	M	HA, LH, C, and V	−	+	+	ITC	1 month
Eng et al. [16]	1993	TCC	71	M	WA, Da, and FD	−	+	+	None	25 days
Eng et al. [16]	1993	TCC	64	M	HA, N, V, A, and PW	−	−	+	ITC and SR	3 months
Hasbini et al. [17]	1997	Undiff bladder Ca	63	M	A and C	X	X	+	None	18 days
Hust and Pfitzer [18]	1982	TCC	52	M	A and Di	+	X	+	None	1 month
Hust and Pfitzer [18]	1982	TCC	66	F	A	X	X	+	None	15 days
Hussein et al. [19]	1991	TCC	60	M	HA	−	+	+	ITC and SR	5 months
Imamura et al. [20]	1997	TCC	71	M	HL, FD, Di, Dp, Da, HA, and NR	+	X	+	Surg resection	< 3 months
Isaka et al. [21]	2002	Small cell NE Ca (SCNC)	67	M	HA and PW	+	+	+	Surgical resection, WBRT, chemotherapy, and Ommaya reservoir	5 months
Kastritis et al. [22]	2011	TCC	67	M	NR, PN, and C	X	X	+	ITC	9 days
Kim et al. [23]	2005	TCC	76	M	C	−	+	+	None	1 month
Lambird and Beerepoot [9]	2018	UC	59	M	HA, Di, HTN, and AM	X	−	+	None	< 1 month
Loizaga iriarte et al. [24]	1999	X	60	M	PW and PN	−	+	X	SCT	< 1 month
Mandell et al. [25]	1985	TCC	59	M	Di, Vt, and HFLD	−	X	+	ITM and SR	X
Molek et al. [26]	2013	Plasmacytoid UC	49	M	CN, Di, A, PW, Sz, and HL	X	X	+	None	10 days
Matsushita et al. [27]	2004	TCC	77	M	NR and PW	X	X	+	None	6 days
Raghavan and Chye [28]	1991	TCC	42	M	HA, C, and Dr	−	X	+	ITC and SR	4 months
Santarossa et al. [29]	1997	TCC	52	F	Sz	−	−	+	ITM and WBRT	9 months
Steg et al. [30]	1993	TCC	68	M	Sz	X	+	−	WBRT	< 1 month
Sugimori et al. [31]	2005	AdenoCa	73	M	HA, A, and SS	−	−	+	None	6 days
Swallow et al. [32]	2015	UC	51	F	Sz, NR, and PW	X	+	−	None	< 1 month
Tadepalli et al. [33]	2011	TCC	47	M	HA and CN	X	+	+	ITM via Ommaya and WBRT	> 9 months
Teyssonneau et al. [34]	2017	UC	63	M	Di and CN	+	+	−	SCT	< 4 years complete response on PET
Tomioka et al. [8]	2021	UC with CIS	45	M	Vt and Da	−	+	+	Dex 6.6 mg IV, WBRT (30 Gy), and pembrolizumab (200 mg)	1 month
Umezawa et al. [7]	2018	UC	66	M	N and PW	X	+	X	Scheduled for WBRT but patient died	14 days
Uncu et al. [35]	2007	TCC	52	M	HA and LH	−	+	+	WBRT and ITM	2 months
Bowen [12]	2000	TCC	46	M	D	−	+	−	ITM and SCT	< 1 month
Bowen [12]	2000	TCC	68	M	C, CDI, and SS	+	X	X	None	< 1 month
Cozzarini et al. [15]	1964	NR	X	X	X	X	X	X	X	NR
Yust-Katz et al. [5]	2013	Signet ring signet cell adenoCa	X	X	X	X	X	X	X	8 weeks
Yust-Katz et al. [5]	2013	TCC	X	X	X	X	X	X	X	29 weeks
Yust-Katz et al. [5]	2013	AdenoCa	X	X	X	X	X	X	X	14 weeks
Yust-Katz et al. [5]	2013	TCC	X	X	X	X	X	X	X	2 weeks
Yust-Katz et al. [5]	2013	TCC	X	X	X	X	X	X	X	6 weeks
Yust-Katz et al. [5]	2013	TCC	X	X	X	X	X	X	X	3 weeks
Yust-Katz et al. [5]	2013	Small oat cell Ca	X	X	X	X	X	X	X	22 week
Yust-Katz et al. [5]	2013	TCC	X	X	X	X	X	X	X	4 weeks
Zada and Chen [36]	2010	TCC	60	M	HA	−	+	+	ITC	NR
This study	2024	UC	63	M	HA, Di, and HL	−	−	+	WBRT	2 months

*Note:* −, negative findings; +, positive findings; x, not reported.

Abbreviations: A, ataxia; AM, adrenal mass; C, confusion; CDI, central diabetes insipidus; CN, cranial nerve palsy; Da, dysarthria; Di, diplopia or vision loss; Dp, dysphagia; Dr, drowsiness; F, female; FD, facial droop; HA, headache; HL, hearing loss; HTN, hypertension; ITM, intrathecal methotrexate; IVS, intravenous steroids; LH, lightheadedness; M, male; N, nausea; NR, nuchal rigidity; PN, polyneuropathy; PW, peripheral weakness; RSc, surgical resection; Sc, syncope; SCT, systemic chemotherapy; SR, spinal radiation; Sz, seizure activity; TCC, transitional cell carcinoma; UC, urothelial carcinoma; V, vomiting; Vt, vertigo; WA, Wernicke aphasia; WBRT, whole brain radiation therapy.

## Data Availability

Data sharing is not applicable to this article as no new data were created or analyzed in this study.
